# Midline Body Actions and Leftward Spatial “Aiming” in Patients with Spatial Neglect

**DOI:** 10.3389/fnhum.2015.00393

**Published:** 2015-07-10

**Authors:** Amit Chaudhari, Kara Pigott, A. M. Barrett

**Affiliations:** ^1^Stroke Rehabilitation Research, Kessler Foundation, West Orange, NJ, USA; ^2^Department of Neurology and Neurosciences, Rutgers-New Jersey Medical School, Newark, NJ, USA; ^3^Department of Neurology, University of Pennsylvania Health System, Philadelphia, PA, USA

**Keywords:** stroke, spatial neglect, motor bias, spatial cognition, visual scanning training, neglect therapy

## Abstract

Spatial motor–intentional “Aiming” bias is a dysfunction in initiation/execution of motor–intentional behavior, resulting in hypokinetic and hypometric leftward movements. Aiming bias may contribute to posture, balance, and movement problems and uniquely account for disability in post-stroke spatial neglect. Body movement may modify and even worsen Aiming errors, but therapy techniques, such as visual scanning training, do not take this into account. Here, we evaluated (1) whether instructing neglect patients to move midline body parts improves their ability to explore left space and (2) whether this has a different impact on different patients. A 68-year-old woman with spatial neglect after a right basal ganglia infarct had difficulty orienting to and identifying left-sided objects. She was prompted with four instructions: “look to the left,” “point with your nose to the left,” “point with your [right] hand to the left,” and “stick out your tongue and point it to the left.” She oriented leftward dramatically better when pointing with the tongue/nose, than she did when pointing with the hand. We then tested nine more consecutive patients with spatial neglect using the same instructions. Only four of them made any orienting errors. Only one patient made >50% errors when pointing with the hand, and she did not benefit from pointing with the tongue/nose. We observed that pointing with the tongue could facilitate left-sided orientation in a stroke survivor with spatial neglect. If midline structures are represented more bilaterally, they may be less affected by Aiming bias. Alternatively, moving the body midline may be more permissive for leftward orienting than moving right body parts. We were not able to replicate this effect in another patient; we suspect that the magnitude of this effect may depend upon the degree to which patients have directional akinesia, spatial Where deficits, or cerebellar/frontal cortical lesions. Future research could examine these hypotheses.

## Introduction

After right brain stroke, over 350,000 people annually each year experience significant functional disability because of an acquired failure to report, respond, or orient to the side of space opposite a brain lesion, a disorder called as spatial neglect (Heilman et al., 1979; Zoccolotti et al., 1989; Buxbaum et al., 2004; Parton et al., 2004; Ringman et al., 2004; Barrett and Burkholder, 2006; Chen et al., 2013). Stroke survivors with spatial neglect, as compared to those with similar severity strokes, experience increased personal and social burden from acute stroke (Buxbaum et al., 2004; Jehkonen et al., 2006) and have poorer rehabilitation outcomes, with slower improvements in walking, bathing, grooming, and toileting, as well as longer hospital stays (Kalra et al., 1997; Gillen et al., 2005; Chen et al., 2015).

Normal spatial processing requires unbiased awareness of the right and left hemispace (perception–attention), an intact internal map (representation), and an ability to plan, initiate and execute symmetric movements (motor-intention) (Barrett and Craver-Lemley, 2008; Shah et al., 2012). The “Where” perceptual–attentional and representational network may mediate directional spatial awareness, vigilance, estimation, and imagery (Coslett et al., 1990; Liu et al., 1992; Na et al., 1998; Buxbaum et al., 2004; Verdon et al., 2010). In contrast, the “Aiming” motor–intentional network may mediate directional action, response inhibition, persistence, and motor/personal self-regulation (Mesulam, 1981; Bisiach et al., 1990; Coslett et al., 1990; Liu et al., 1992; Ladavas et al., 1993; Na et al., 1998; Rossit et al., 2009; Verdon et al., 2010; Vossel et al., 2010; Goedert et al., 2014).

Patients with strokes affecting the motor–intentional network present with Aiming spatial bias, which is formally defined as a dysfunction of the brain networks enabling initiation and execution of motor–intentional behavior (Adair and Barrett, 2008). This body-based disorder causes patients to make errors of hypokinetic movements (movements that fail to initiate because of muscular rigidity or other factors) and hypometric movements (movements that are initiated but fall short of the intended goal) toward the left side of space (Na et al., 1998; Heilman, 2004; Riestra and Barrett, 2013), which are likely to contribute to postural, balance, and movement problems in the acute stroke setting. Aiming spatial bias may be uniquely functionally relevant (Goedert et al., 2012), and may be associated with damage to subcortical–cortical, and/or frontal–cortical brain networks (Mesulam, 1981; Bisiach et al., 1990; Coslett et al., 1990; Liu et al., 1992; Ladavas et al., 1993; Na et al., 1998; Rossit et al., 2009; Verdon et al., 2010; Vossel et al., 2010; Goedert et al., 2014).

A standard approach to spatial neglect rehabilitation is based upon visual scanning training (Weinberg et al., 1977), in which participants are cued to orient toward the contralesional side of space. In patients with spatial neglect, spontaneous saccadic eye movements or head movements are restricted to the ipsilesional visual hemifield and may rarely cross midline (Zoltan, 1996; Chaikin, 2007). Visual scanning training expands these movements into the contralesional space by repeatedly using cues, such as “point your right (unimpaired) hand to the left side” or “look to the left.” Recent studies have shown that visual scanning exercises increase perceptual processing (Where networks) in neglect patients, thereby leading to better visual function and ability to perform activities of daily living (van Wyk et al., 2014).

However, it is possible that the brain mechanisms governing axial (midline) versus appendicular (paired structure or limb) movements might interact with scanning and body movement cuing to increase or decrease the effectiveness of Weinberg et al. (1977) method. Geschwind (1965, 1975) argued that the pyramidal motor system is involved in discrete movements of the appendages, like the ones most commonly used for cueing in visual scanning training. In contrast, a non-pyramidal motor system, which may engage a more bilaterally distributed network, may be more capable of assisting with orienting in people with spatial Aiming deficits caused by spatial neglect (Poeck et al., 1982). Specifically, we hypothesize that visual scanning therapy may be more effective when using structures innervated by the non-pyramidal motor system, such as the head and trunk. These midline structures would engage brain mechanisms less impaired by right parietal lobe stroke and help patients plan, initiate, and execute movements in the contralesional hemispace. Although one study examined the beneficial effect of a midline movement, trunk rotation (Wiart et al., 1997), as part of scanning training, specific methods of screening for and addressing spatial Aiming errors during neglect rehabilitation are not widely available.

We observed that a patient with spatial neglect, who had problems orienting leftward to identify objects on her left, had difficulty using her right, ipsilesional hand (not obviously affected by paralysis) to point leftward. Based on the above hypotheses, we wished to learn whether instructing the patient to move midline body structures would result in better leftward orienting, than would instructing her to initiate leftward pointing movements with the right hand. First, we conducted a single case study to test a specific hypothesis and then, on the basis of the results obtained on this patient, we performed a preliminary group study to explore the same hypothesis in a larger sample of patients. Our study was not intended to evaluate efficacy of a therapy, which can be better performed in group studies, but rather to formulate a paradigm for future and expanded evaluation (proof of concept).

In the present study, we compared instructions to move midline (nose, tongue) versus non-midline (eyes, hand), body parts to test their effect on leftward orienting, and we predicted that midline body parts would facilitate more leftward orienting.

## Experiment 1: Single Case Study

### Subject

We tested a single subject with spatial neglect after stroke, who had difficulty in using right hand movements to facilitate leftward orientation and to evaluate our above hypothesis. A 68-year-old female presented with right-sided facial weakness and left arm and left leg weakness 3 weeks prior to volunteering for our study. She was brought into the emergency room, where she was found to have atrial fibrillation and a right basal ganglion acute infarct with no bleeding. Antiplatelet (clopidogrel), anti-hypertension (metoprolol, ezetimibem, furosemide), and anticoagulation (warfarin) were started immediately and continued past discharge. See Table 1 for demographic details about the participant.

**Table 1:** **Demographics and clinical information about all participants**.

Experiment	Patient	Age	Sex	Years of education	Stroke side	Race	Primary hand	Days post-stroke	BIT score	CBS score			Barthel index	MMSE score
										Total	CBS-ME	CBS-PA	
Experiment 1	1	68	F	12	Right	African-American	Right	21	28	24	11/12	13/18	10	16
Experiment 2	1	79	F	12	Right	Caucasian	Right	17	109	9	6/12	3/18	80	23
	2	83	F	12	Right	Caucasian	Right	21	14	28	12/12	16/18	20	11
	3	76	M	16	Right	Caucasian	Right	22	30	26	12/12	14/18	25	25
	4	82	M	5	Right	African-American	Right	20	14	24	10/12	14/18	10	18
	5	64	M	16	Right	Asian	Right	17	14	25	11/12	14/18	40	19
	6	81	M	16	Right	Caucasian	Right	16	23	22	10/12	12/18	15	16
	7	56	M	11	Right	Caucasian	Right	17	8	26	11/12	15/18	0	24
	8	82	F	12	Right	Haitian	Ambidextrous	19	40	20	10/12	10/18	60	18
	9	58	F	12	Right	Caucasian	Right	44	22	21	11/12	10/18	10	21

**Figure 1:**
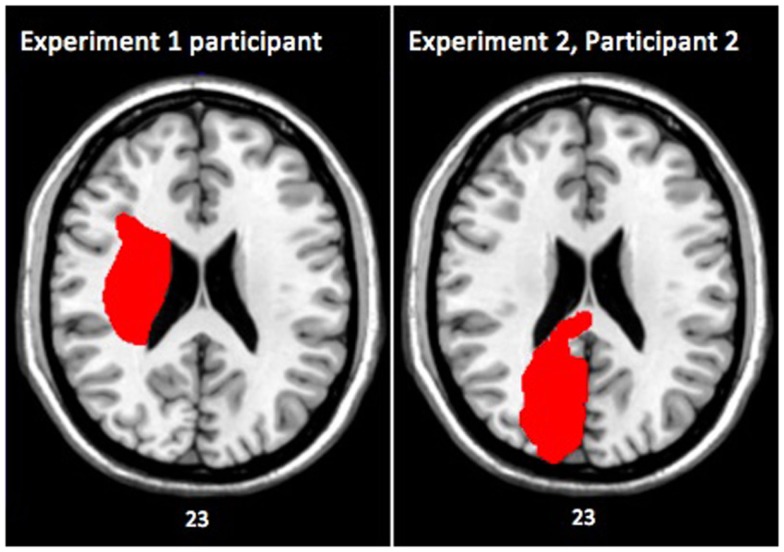
**Axial slices of brain lesions from the single case participant in Experiment 1 (left), and from participant 2 in Experiment 2 (right)**. Both images are shown in MNI space with the left side of the image representing the right side of the patient’s brain. The numbers underneath the images denote the *z*-coordinates of the MNI template.

The patient was awake, alert, and oriented ×3. She had a right facial droop and rightward gazing preference. The evaluation of motor strength was conducted according to guidelines from O’Brien (2010). Strength was 0/5 in the left upper and lower extremities, and 5/5 in the right upper and lower extremities. Sensation was not tested. Computed tomographic scanning revealed infarction in the right putamen, thalamus, and the right internal and external capsules, with possible involvement of the right caudate and right temporal lobe (see Figure 1).

Prior to starting, the patient gave written informed consent and all data were obtained in compliance with the Kessler Foundation IRB regulations. She met criteria for severe spatial neglect [behavioral inattention test (BIT) (Wilson et al., 1987) conventional score = 28/146, and Catherine Bergego Scale (CBS) (Azouvi et al., 2003) score = 24/30]. In addition, she did not have visual problems including blindness, glaucoma, or others. She did not have a history of a serious brain condition other than stroke, and did not have Alzheimer’s disease or dementia or a history of psychiatric hospitalizations. She was enrolled in the study 3 weeks post-stroke.

Based on a method developed by our lab, we used the CBS to determine the Aiming and Where scores of our participant. Goedert et al. (2012) previously conducted a factor analysis on the CBS and found that motor–intentional behavior was directly related to four items: limb awareness, dressing, navigation, and collisions. Thus, the total Aiming bias of an individual could be calculated as the total score on these four CBS motor–intentional (CBS-ME) items, which ranges from 0 to 12 with higher scores indicating worse performance (Goedert et al., 2012; Shah et al., 2012). The other perceptual–attentional (CBS-PA) items related to Where spatial bias, whose total score ranged from 0 to 18 with higher scores indicating worse performance (Goedert et al., 2012; Shah et al., 2012). Our participant scored 11/12 (91.7%) on the CBS-ME and 13/18 (72.2%) on the CBS-PA, indicating that she had a primary Aiming with an underlying Where spatial bias.

### Methods

**Figure 2:**
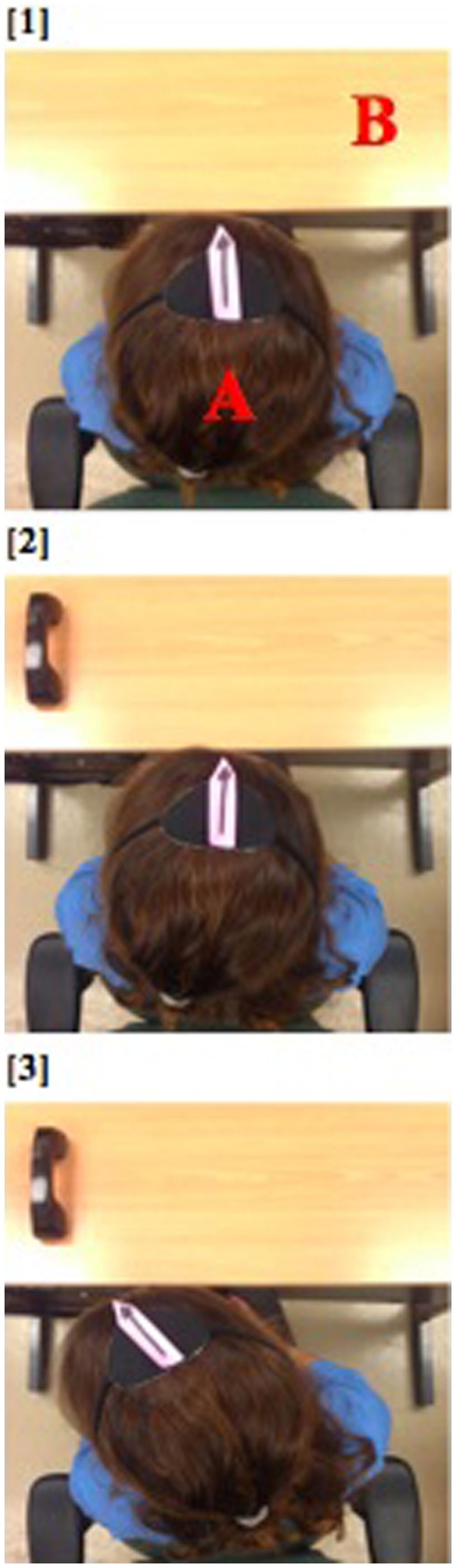
**Image 1 shows the experimental apparatus – letter “B” denotes the workspace, while letter “A” shows the participant’s head with a pink arrow pointing to his nose**. The participant’s response is incorrect if there is no/non-leftward movement of the orange arrow after placement of the stimulus (phone); see image 2. A response is correct when the orange arrow moves toward the stimulus (phone); see image 3.

We noted in this patient that standard visual scanning therapy was challenging, as she had great difficulty using her right, less affected hand to point leftward, and her therapists used this instruction frequently. To investigate whether midline actions improved her ability to orient leftward, after obtaining informed consent, we had her to wear a cap with an arrow pointing toward her nose. A camera, placed directly above, recorded her ability to orient leftward (see Figure 2) as she was asked to identify an object on her left (pen, keys, or phone; see Figure 2) using either midline movement instructions: “point with your nose to the left” and “stick out your tongue and point it to the left” or axial movement instructions: “look to the left,” and “point with your [right] hand to the left” (12 trials per instruction). We did not specifically describe any difference between the conditions, but simply instructed that she point in the manner specified. However, the examiner was aware of the study hypothesis. A second experimenter who viewed the videotaped performance scored each trial.

A trial was scored as 1 (correct) if the arrow on the patient’s cap moved leftward beyond the midline after the instruction. Any leftward response going past midline and targeted toward the object during the time from the instruction to the patient either identifying the object or saying “I don’t see it” was counted. The patient’s ability to correctly identify the actual object was not taken into consideration. A score of 0 (incorrect) was given to trails where the arrow on the patient’s cap failed to move leftward beyond the midline.

A total of 12 trials were conducted for each instruction before moving on to the next. The sequence of instructions was randomized.

Clinical images (CT or MRI) were obtained for our participant. Using MRICron, her lesion was first mapped on the transverse plane of her own brain, and then realigned with stereotaxic Montreal Neurological Institute (MNI) space (Rorden and Brett, 2000; de Haan and Karnath, 2013). Note: all MRI images are presented where the left side of the figure represents the right side of the participant’s brain.

### Results of experiment 1

Our patient correctly named left-sided objects in 42% of trials with looking, 50% of trials with nose-pointing, 0% of trials with hand pointing, and 100% of trials with tongue-pointing (see Table 2). A χ^2^ test showed that her performance significantly improved when instructed to move midline (nose, tongue) versus paired or appendicular (eyes, hand), body parts (χ^2^ = 54.56, *p* < 0.0001). In particular, her ability to orient leftward when she moved her tongue left appeared remarkably better than her ability to orient leftward when asked to move her hand left. This suggests that nose and tongue movements may be less affected by spatial neglect than are eye and hand movements. It also suggests that an alternate method of administering visual scanning training, instructing the patient to move her tongue to the left, might increase training effectiveness.

**Table 2:** **Percentage of correct responses in each instruction type**.

Experiment	Participant	Midline		Non-midline		Chi square *p*-value
		Nose (%)	Tongue (%)	Eyes (%)	Hand (%)	
Experiment 1	1	50	100	42	0	<0.0001
Experiment 2	1	100.0	100.0	100.0	100.0	
	2	13.3	13.3	26.7	13.3	
	3	100.0	100.0	100.0	100.0	
	4	100.0	100.0	100.0	100.0	
	5	100.0	100.0	100.0	100.0	
	6	93.3	93.3	80.0	93.3	
	7	66.7	33.3	26.7	86.7	
	8	100.0	100.0	100.0	100.0	
	9	73.3	93.3	73.3	80.0	
	Average scores of participants 2, 6, 7, and 9	62	58	52	68	0.8495

## Experiment 2: Case Series of Consecutive Patients with Spatial Neglect

If there is dissociation between the ability to use eye, hand, nose, and tongue movements to facilitate leftward orienting, this effect might be robust in people with all kinds of spatial neglect characteristics. However, it is also possible that, as our group has repeatedly observed and discussed, the specific deficit characteristics of the patient’s spatial neglect syndrome may interact with the mechanisms of the intervention, resulting in different degrees of performance change in different patients (Barrett and Burkholder, 2006; Barrett et al., 2012; Goedert et al., 2014). To test whether a uniform or variable effect might be expected in groups of patients with spatial neglect, we evaluated nine consecutive stroke survivors.

### Subjects

Nine consecutive patients (4 female, age 75 ± 11 years) with spatial neglect after an acute right hemispheric stroke (mean time post-stroke = 2.8 weeks) were recruited for the study (see Table 1 for demographics). All participants gave written informed consent and all data were obtained in compliance with the Kessler Foundation IRB regulations. We included patients who (1) were admitted to the rehabilitation hospital between the ages of 18 and 100 years, (2) suffered from a right hemispheric stroke and had spatial neglect, (3) had a stroke between 5 days and 4 months prior to enrollment into the study, (4) were not blind in one or both eyes, (5) did not have uncontrolled glaucoma, (6) did not have a history of a serious brain condition other than stroke, (7) did not have Alzheimer’s disease or dementia, and (8) did not have a history of psychiatric hospitalizations.

We used the same methods as in Experiment 1 to evaluate for each participants Aiming and Where spatial bias. Our participants had a mean CBS-ME score of 86.1 ± 15.0%, and a CBS-PA score of 66.7 ± 22.0%. Each patient’s individual scores are summarized in Table 1.

**Figure 3:**
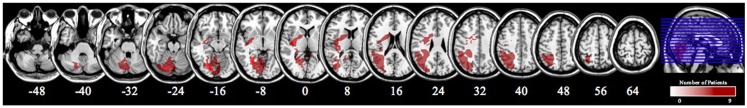
**An overlap of the brain lesions of all nine patients in Experiment 2**. All lesions were drawn using MRICron Version 6. Lesions were mapped onto MNI space and smoothed to 3 mm using FWHM. The numbers underneath the images denote the *z*-coordinates of the MNI template.

Clinical images (CT or MRI) were obtained for all participants. Using MRICron, individual lesions were first mapped on the transverse plane of respective images, and then realigned with stereotaxic MNI space to overlap them on standard brain templates (Rorden and Brett, 2000; de Haan and Karnath, 2013). Figure 3 illustrates the overall lesion location and size for all participants. Note: all MRI images are presented where the left side of the figure represents the right side of the participant’s brain.

A review of the brain scans for patients did not suggest two disorders (large vessel stroke plus small-vessel, distributed ischemic leukoencephalopathy). In addition, we analyzed all of the patients’ medical history and physical exam reports, as well as radiology reports, to ensure that their primary lesions were cerebrovascular events in the right hemisphere and there was no specific history suggestive of other progressive leukoencephalopathy.

### Methods

To evaluate whether cuing movement of midline versus paired body structures improved leftward orienting more than cuing hand movements, we used the same protocol devised for the patient described in Experiment 1, except that we used 15 trials for each of 4 conditions: “point with your nose to the left,” and “stick out your tongue and point it to the left” (midline) or “look to the left,” and “point with your [right] hand to the left” (paired/limb).

### Results of experiment 2

All nine stroke patients in Experiment 2 made extrapersonal spatial errors on the BIT and CBS. However, most (6/9) of them were 100% able to orient toward objects in left space under all conditions of this protocol, which was considerably easier than the items evaluated in the BIT. Therefore, we were not able to evaluate these patients for improvement in leftward orienting with midline cuing; their data were not considered further. Of the four patients who made errors, only one made many leftward orienting errors when she was instructed to use right hand pointing (subject 2; 13.3% correct when pointing leftward with the right hand). In this patient, using the nose and tongue did not seem to improve her ability to orient leftward (same accuracy rate as when pointing with the hand) and instruction to look leftward improved her leftward orienting only slightly (26.7% correct). This patient had a similar spatial neglect symptom profile to the patient in Experiment 1, with similar severity on the BIT and CBS and similar numbers of errors on CBS motor-exploratory items (Goedert et al., 2012). However, her brain lesion on imaging had different characteristics, with acute lesions in the right occipital lobe and left cerebellum, mild periventricular white matter changes consistent with chronic microvascular ischemic disease, and old bilateral basal ganglia lacunes noted.

Of the other three patients who made errors, none made the most errors when supporting leftward orienting by attempting to point leftward with the right hand. Two patients had greatest difficulty looking leftward (patients 6 and 7: 80 and 26.7% accurate when looking leftward versus 93% accurate under all other conditions for patient 6 and 86.7% accurate pointing with the hand, 66.7% accurate pointing with the nose, and 33% accurate pointing with the tongue for patient 7). The last patient had the greatest difficulty with looking or pointing the nose leftward (73.3% accurate) while pointing with the tongue or hand was more accurate (tongue 93.3%; hand 80%).

A χ^2^ test in the group of four patients who made errors, evaluating for differences in response between instructions to use midline versus paired/limb body movements, did not reach significance (χ^2^ = 0.8, *p* = 0.85, n.s).

## Discussion

We observed that we could facilitate left-sided orientation and object identification by instructing a stroke survivor with spatial neglect to point with the tongue, instead of using the hand. This is of relevance because some forms of visual “anchoring” or scanning training emphasize using the hand to point, feel, or locate leftward landmarks; others emphasize “looking leftward,” which is an instruction to move the eyes. When asked to point leftward with her tongue, the patient was able to orient leftward during 100% of trials, as contrasted with 0% of trials when pointing with the hand, and 42% of trials when she looked leftward. Our results suggest that using midline body parts may facilitate the impact of therapeutic interventions, such as visual scanning training, in patients who are have primary Aiming spatial bias and fail to respond with traditional techniques using the hand or eyes.

Unfortunately, when we evaluated more patients with spatial neglect, we did not have the opportunity to replicate the effects of instructions for body movements that we observed in Experiment 1. While all nine stroke patients in Experiment 2 made extrapersonal spatial errors on the BIT and CBS, only four of them made any errors in identifying objects on this protocol, which was considerably easier than the items evaluated in the BIT. Only one of the four people failed to orient leftward more than 50% of the time when using the right hand to point left. Although our sample, drawn from patients admitted to inpatient rehabilitation, may not be representative, this suggests that leftward directional hypokinesia affecting the hand may occur in over 20% of patients with moderate–severe stroke and spatial neglect. Although further research is needed to confirm these proportions, the commonly used techniques of using the left hand to point, explore or self-cue leftward, or encouraging the patient to look leftward in respond to verbal exhortations, were not effective either in this patient, or the patient in Experiment 1. Visual scanning training incorporates multiple factors, but it is possible that poor response to instructed hand and eye movements is part of the reason why, in previous groups of treated patients, this method required several weeks before subjects showed improvements (Weinberg et al., 1977; Antonucci et al., 1995). The second patient with difficulty pointing the right hand leftward, unfortunately, did not orient leftward more accurately after being provided the instruction to point leftward with the tongue, as did the patient in Experiment 1.

The efficacy of cuing patients to move parts of the body in order to facilitate leftward orienting in patients with spatial neglect may be influenced by several patient-specific factors. As we suggested, different brain mechanisms may govern axial (midline) versus appendicular (limb) movements. If bilateral brain systems activate midline body parts, this might make these body parts more effective to cue orienting, because brain systems that are not damaged by a right brain stroke could be recruited to assist accurate orienting movements. In particular, the patient in Experiment 1 oriented leftward very well when pointing with the tongue, an axial body structure.

If we regard the tongue as an axial body part and the eyes as a paired, non-midline body part, then our results are consistent with potential facilitation of leftward orienting with midline body part movements in the patient presented in Experiment 1. However, this hypothesis does not explain the failure of tongue movements to activate leftward orienting in subject 2 in Experiment 2, or the patterns of performance in patients 6, 7, and 9. Poeck et al. (1982) pointed out that movements of the tongue and midline oral–pharyngeal body parts are frequently affected by cognitive-motor deficits that also affect the limbs (limb apraxia). These authors also pointed out that Geschwind’s original (Geschwind, 1965) hypothesis grouped the paired eye movements with axial movements as midline body actions; therefore, it may not be surprising that exhorting leftward eye movements failed to produce any dramatically beneficial effect on leftward orienting in the impaired patients in Experiment 2.

Another possible way of explaining the effect of tongue movements on leftward orienting in the patient presented in Experiment 1 is to propose that the ability of body movements to facilitate leftward orienting may depend upon their position on the body. Robertson and North (1993) conducted a series of three experiments in which they showed that active movements of left body parts in left hemispace led to maximal improvements in neglect performance. Movements of the right limbs did not produce similar results. Thus, a therapeutic effect of moving a midline body part relative to the hand may be mediated by its more leftward position on the body. These results are supported by McCarthy et al. (2002) who showed that even imagined movements of the right hand had an adverse effect on performance in spatial neglect. Unfortunately, we did not explicitly test this hypothesis by instructing patients to move their left hand, arm, or leg (Fong et al., 2007). However, because the left eye is further leftward on the body than the tongue or nose, the slight improvement seen with leftward eye movements in subject 2 in Experiment 2, who was most similar to the patient in Experiment 1, might be explained by this effect. Future experiments could evaluate more systematically how left-, midline-, and right-sided body parts affect leftward orienting.

It is possible that the beneficial effect of tongue movements on leftward orienting may depend upon the degree to which patients have directional akinesia and body-centered spatial motor Aiming bias. Unfortunately, we did not directly measure directional akinesia in these patients with spatial neglect, such as with equipment that can fractionate perceptual–attentional Where versus motor–intentional Aiming spatial errors (Barrett and Burkholder, 2006; Garza et al., 2008; Chen et al., 2011). Both the patient reported in Experiment 1 and subject 2 in Experiment 2 performed abnormally on motor-exploratory items from the CBS (Goedert et al., 2012). However, no body part cued resulted in >50% leftward orienting in subject 2, Experiment 2. We do not know whether this patient may have had much greater leftward hypokinesia, or whether the presence of an additional perceptual–attentional Where spatial bias may have influenced the success of a cuing intervention. Future studies investigating that the efficacy of visual scanning therapy in patients with spatial neglect can greatly benefit from evaluating the above measures at baseline.

Lastly, it is possible that the neuroanatomic networks affected in the patients in our study explained the difference between the effect of tongue movements in the patient in Experiment 1 and the patients in Experiment 2, in particular, subject 2. Lesions in basal ganglia-frontal cortical networks may play a permissive role in body-motor cuing, as we suggested they might in prism adaptation training (Chen et al., 2014). Alternatively, lesions affecting the cerebellum, as in subject 2 and two other subjects in Experiment 2, may affect tongue and oral movements (Marien et al., 2013; Akin et al., 2014). It is possible that damage to the cerebellum may also lead to deficits in the non-pyramidal motor system, which controls head and trunk rotation. However, Stoodley and others have shown that the left cerebellar hemisphere is most involved in spatial functioning (Stoodley et al., 2012). The participants in Experiment 2 had very minor, if any, lesions in the left cerebellum; hence, making the possibility of non-pyramidal involvement less likely. Future research could examine these, and the above hypotheses.

While our results were observed primarily in one person at this stage, we feel that they significantly augment the clinical principles used for treatment, could encourage clinicians to pursue alternate, low-risk techniques in administering therapy, and provide credibility for future theoretical investigations. Future experiments could increase task difficulty by asking patients to perform neglect assessments, such as cancelation tasks or the line bisection test before and after each 15-trial instruction set. More patients with neglect would be expected to make some errors at baseline on these tasks as opposed to the one used in this study, thereby allowing for a more sensitive analysis of change in Aiming spatial bias with instructions using midline body parts. Future studies could also more formally evaluate the presence of other disorders, such as leukoencephalopathy.

## Author Contributions

The three authors are justifiably credited with authorship, according to the authorship criteria. In detail: AC – analysis and interpretation of data, drafting of the manuscript, final approval given; KP – acquisition of data, analysis and interpretation of data, final approval given; AB – conception, design, analysis and interpretation of data, critical revision of manuscript, final approval given.

## Conflict of Interest Statement

Mr. Amit Chaudhari has nothing to disclose. Ms. Kara Pigott has nothing to disclose. Dr. A. M. Barrett has received honoraria for online textbook chapters for eMedicine/WebMD. Dr. A. M. Barrett has received research support from Kessler Foundation, National Institutes of Health, the Wallerstein Foundation for Geriatric Improvement, the Department of Education/NIDRR.
